# Enhancement of Nano-Biopolymer Antibacterial Activity by Pulsed Electric Fields

**DOI:** 10.3390/polym13111869

**Published:** 2021-06-04

**Authors:** Mai. I. El-Kaliuoby, Motaz Amer, Nader Shehata

**Affiliations:** 1 Faculty of Education, Alexandria University, Alexandria 21544, Egypt; mai.ismail@alexu.edu.eg; 2 Basic and Applied Science Institute, College of Engineering Arab Academy for Science, Technology and Maritime Transports, Alexandria 21544, Egypt; motaz.amer@aast.edu; 3 The Bradley Department of Electrical and Computer Engineering, Virginia Tech, Blacksburg, VA 24061, USA; 4 Department of Engineering Mathematics and Physics, Faculty of Engineering, Alexandria University, Alexandria 21544, Egypt; 5 Kuwait College of Science and Technology, Doha Area, 7th Ring Road, Safat 13133, Kuwait; 6 Utah Science Technology and Research (USTAR) Bio-Innovation Center, Utah State University, Logan, UT 84341, USA

**Keywords:** natural biopolymers, nano-chitosan, pulsed electric fields, antibacterial, *P. aeruginosa*, *S. aureus*, wound healing

## Abstract

Chronic wounds are commonly colonized with bacteria in a way that prevents full healing process and capacity for repair. Nano-chitosan, a biodegradable and nontoxic biopolymer, has shown bacteriostatic activity against a wide spectrum of bacteria. Effectively, pulsed electromagnetic fields are shown to have both wound healing enhancement and antibacterial activity. This work aimed to combine the use of nano-chitosan and exposure to a pulsed electric field to overcome two common types of infectious bacteria, namely *P. aeruginosa* and *S. aureus*. Here, bacteria growing rate, growth kinetics and cell cytotoxicity (levels of lactate dehydrogenase, protein leakage and nucleic acid leakage) were investigated. Our findings confirmed the maximum antibacterial synergistic combination of nano-chitosan and exposure against *P. aeruginosa* than using each one alone. It is presumed that the exposure has influenced bacteria membrane charge distribution in a manner that allowed more chitosan to anchor the surface and enter inside the cell. Significantly, cell cytotoxicity substantiates high enzymatic levels as a result of cell membrane disintegration. In conclusion, exposure to pulsed electromagnetic fields has a synergistic antibacterial effect against *S. aureus* and *P. aeruginosa* with maximum inhibitory effect for the last one. Extensive work should be done to evaluate the combination against different bacteria types to get general conclusive results. The ability of using pulsed electromagnetic fields as a wound healing accelerator and antibacterial cofactor has been proved, but in vivo experimental work in the future to verify the use of such a new combination against infectious wounds and to determine optimum treatment conditions is a must.

## 1. Introduction

Recently, the integration of an array of antimicrobial agents against resistant bacteria has opened great interest in overcoming the spread use of antibiotics. Nanoparticles have opened new areas for new antibacterial materials with novel properties and advanced characteristics [1,2,3]. Growing interest has arisen in chitosan biopolymer nanoparticles (ChNPs) as a biodegradable and nontoxic material with considerable bio-applications [4]. It has demonstrated a broad antibacterial activity against Gram-positive and Gram-negative bacteria [5] due to its unique biological characteristics. One of the most proper mechanisms of ChNPs’ antibacterial action is based on the probability of electrostatic communication with either the bacterial cellular wall or the cell membrane. This electrostatic communication occurs between the positive charges of amino groups of glucosamine with the negative charge of the cell membranes of bacteria [6]. This interaction leads to variations in the surface of the cell, leading in turn to modifications in the membrane permeability which sequentially facilitate osmotic imbalance and efflux of intracellular substances that result in cell death [7].

To another extent, the consideration of ChNPs as bactericidal was limited recently to biostatic. Instead, the need for external cofactors to enhance its bactericidal impact has become a must [8,9,10]. Recently, the combination of using ChNPs and a laser diode as cofactors against *E. coli* strains was studied. The obtained results indicated the ability of the laser diode to excite the ChNPs and hence cause photothermal lysis of *E. coli* cells. The synergism between using ChNPs and a laser diode increases the bacteria inhibition process and causes a more lethal impact than using ChNPs alone [11]. In another way, the antibacterial effect of nano-silver particles against *E. coli* was studied in combination with ultraviolet light by Zazouli et al. [12]. The obtained results confirmed the intensified disinfection ability of nanoparticles combined with exposure to ultraviolet light which was attributed to the production of electrons, active holes and active radicals by the irradiation. The great interest during the last ten years and the series of questions raised about the negative and/or positive biological effects of electromagnetic waves, especially in the extremely low frequency (ELF) range, make this an open-ended research area. In this regard, the influence of ELF pulsed electric fields (PEFs) on bacteria as co-stressors showed remarkable effects on treatment bio-availability and increased the antibacterial susceptibility as reported elsewhere [13,14,15,16,17,18,19,20]. A nontraditional antibacterial treatment was performed by using an array of nanofiber mats together with exposure to electromagnetic waves. The applied electromagnetic waves (EMWs) were shown to be a synergistic co-factor in killing bacteria even at low nanofiber mat concentrations [21]. In a different way, exposure to a magnetic field of 1.8 mT for 20 min was adopted to develop the kinetics of dentin/enamel re-mineralization in combination with nano-chitosan gels. The results established the applicability of using a magnetic field as an accelerator for tooth remineralization [22]. In addition to the ability of PEF to decrease the bacteria growth rates it also accelerates the wound healing process [23,24]. PEF efficiently improves of wound healing kinetics by redistributing and activating important membrane receptors, manipulation of ion transportation at the wound and the ability to direct cell migration.

One of the most serious problems in wound treatment is healing of chronic wounds that fail to heal through the body’s natural healing processes. Most causes of delayed healing in chronic wounds are, but not limited to, microbial infection, poor blood circulation and other severe impaired healing processes such as lack of epithelial migration, angiogenesis, and cell proliferation. Wound infection occurs due to a disturbed host–bacteria equilibrium process in a traumatized tissue environment which favors bacteria and leads to inhibition of the healing process [25]. In addition, it is important to bear in mind that the microbial contamination may not be only on the surface but also deep in the wound tissue, that could be complicated by the presence of biofilms and/or antibiotic resistance. As a result, external exposure to PEF as a cofactor with antibacterial agents to enhance the wound healing process and accelerate tissue repair are required.

To address the current challenges, we proposed the combine used of ChNPs and ELF-PEFs as an antimicrobial and wound healing accelerator. To demonstrate such a new combination, the antibacterial effects of ChNPs and ELF-PEFs were tested against Gram positive and negative bacteria models as an example of highly infectious wounds. In chronic wounds, *Staphylococcus aureus* (*S. aureus*) and *Pseudomonas aeruginosa* (*P. aeruginosa*) colonize about 93.5% and 52.2% of patients with chronic leg ulcers, respectively [26,27,28,29]. The bacterium *P. aeruginosa* has the ability to establish itself in biofilms [30,31]. These biofilms are complex structures that act as efficient barriers against antibiotics and thus the host immune system weakens the bacterial infections and wounds become more difficult to heal [32]. On the other hand, *S. aureus* is an antibiotic-resistant pathogenic bacterium, which enhances infections by producing factors that cause bacteremia, endocarditis, skin tissue infections, and also hospital-acquired infections [33,34]. Therefore, the antibacterial effects of ChNPs alone, ELF-PEFs alone and combinations of them were tested against *P. aeruginosa* and *S. aureus*. The antibacterial effects were determined by studying the bacterial growing curves, growth kinetics and cytotoxicity. Evaluation of bacteria cytotoxicity was obtained by monitoring levels of lactate dehydrogenase (LDH), protein leakage and nucleic acid leakage for both strains.

## 2. Materials and Methods

### 2.1. Electric Field Exposure System

The exposure was achieved by using two parallel plate capacitor systems connected to a DC power supply locally manufactured at the Physics Lab of the Alexandria University Faculty of Science (Alexandira, Egypt). The input DC voltage of 6 V was directed to the exposure system through an electronic switching device of 50% duty cycle to maintain interrupted currents at different frequencies. The interrupted currents are then directed into a DC/DC voltage converter to amplify the voltage up to 400 V. For exposure purposes, the output voltages were conducted through rectangular aluminium plates of 20 × 10 cm^2^ and separated by 10 cm. The supernatant samples were placed at the midline between the two plates and electric field was adapted between the two plates and confirmed by using a field meter type Tri-field^®^ TF2 EMF meter (AlphaLab, Salt Lake City, UT, USA) of accuracy of ±5%. The measured peak electric field intensity was found to be 3.70 ± 0.18 kV/m as recorded at the exposure point located in the central axis of the supernatant glass tubes. The pulse shape was displayed using a GOS-620 type oscilloscope (GW Instek, New Taipei City, Taiwan) and found to be squared pulses. The exposure time was adjusted to be 30 min for all experimental conditions. For controlling the exposure conditions, the supernatant glass tubes were placed on top of a nonconductive stand and the temperature was adjusted to be in the 27 °C range as recorded by using an electronic thermometer type SP Bel-Art, H-B DURAC Calibrated Electronic Thermometer (Durac, Beijing, China). It is worth clarifying that there is no conflict between our exposure facility and the safe limits that have been addressed by the International Commission on Non-Ionizing Radiation Protection.

### 2.2. Nano-Chitosan Characterizations

**Figure 1:**
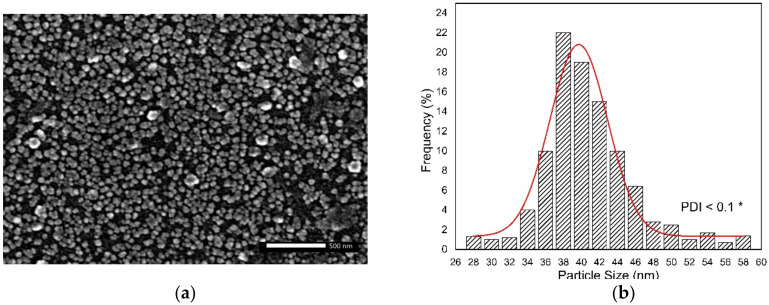
(**a**) SEM image of ChNPs indicates grains of particles without agglomeration, and (**b**) histogram of particle sizes distribution shows average size of ChNPs to be 39.68 nm. * Polydispersity Index (PDI).

The chitosan nanoparticles (ChNPs) used in the present work were purchased from Alpha Chemika Co. (Mumbai, India). Their shape and size were confirmed by scanning electron microscopy (SEM) as shown in Figure 1. The samples were sputter-coated with gold and the morphology observed at an acceleration voltage of 20 kV with a highest magnification of 40,000× *g*. Figure 1a showed grains of ChNPs without agglomeration and the size distribution profile indicated a particle size range between 28.61 nm to 57.84 nm and an average size of about 39.68 ± 0.22 nm, as presented in Figure 1b.

### 2.3. Bacterial Strains and Replications

In this work, we used two bacterial strains. One is *P. aeruginosa*-ATCC 27,853 as a Gram negative model bacterium and the other one is *S. aureus*-ATCC 25,923 as a Gram positive model organism. It should be stated that the Gram positive bacterial strain of *S. aureus* was a methicillin-susceptible MSSA. The bacterial strains of *P. aeruginosa* and *S. aureus* were plated on nutrient agar plates for further antibacterial tests. The growing conditions were kept constant for both strains during the whole experiment in which approximately 10^5^ colony forming units (CFU) from each pathogen were inoculated on agar plates and allowed for incubation 24 h at 37 °C. Subcultures from each bacterial strain were obtained by periodic inoculation of several colonies to maintain fresh strains.

### 2.4. Bacterial Growth Characteristics and Antibacterial Impacts

The bacteriostatic impact of ChNPs against bacterial strains was determined by using a plate count technique in which both positive and negative strains (0.5 McFarland ≡ 1.5 × 10^8^ CFU/mL) were inoculated in broth media and exposed to serial concentrations of ChNPs. In advance, ChNPs stock solution was prepared by adding chitosan ChNPs in acetic acid solution (1% *w/v*) and stirring for 12 h to dissolve the powder. The final concentration of the ChNPs stock was 1% (*w/v*) and it was diluted as needed using deionized distilled water. Serial dilutions starting from 500 mg/mL were reduced by a factor of 1.5 to a concentration of 1.14 mg/mL. It is worth clarifying that ChNPs powder was sterilized by exposure to ultraviolet radiation and broth media were sterilized by autoclaving at a temperature up to 121 °C. Furthermore, inoculations of 100 µL from each bacterial strain were mixed with 100 µL of ChNPs for all serial dilutions and left to incubate 24 h at 37 °C. The concentration at which no visible growth of bacteria in broth media was considered as minimum inhibitory concentration (MIC) and the concentration at which three or less colonies were observed (>99.9% killed) after inoculation in agar plates was considered as minimum bactericidal concentration (MBC) [35]. To further select concentrations less than the MIC levels of ChNPs were supplemented to both bacterial strains (positive control samples) in broth media and exposed to PEF ((3.70 ± 0.18) kV/m-30 min) at 0.7 Hz and 20 Hz for *P. aeruginosa* and *S. aureus* respectively, as reported by Fadel et al. [36] and El-Kaliuoby et al. [37]. The viability of bacterial cells is calculated as a percentage relative to samples that didn’t receive any treatment (negative control ones): viability% = [(average count of control − average count of treated)/average count of control] × 100. In this regard, the growth characteristics were studied by observing cell populations as monitored by turbidity measured in terms of optical density at 600 nm (OD_600_). [37] The growing characteristics were presented as growth curves plotted between OD_600_ and incubation time to represent the effect of exposure in bacterial growth phases. Furthermore, the growth dynamics were obtained by analyzing the growing curves and calculating their arbitrary rate constants as follows:

Arbitrary growth rate constant = 1/t ln (N/N_0_)

where N is the bacterial cell count at time (t) and N_0_ the initial cell count. In proceeding, the arbitrary rate constants were trended and plotted versus ChNPs concentrations (0–100 mg/mL) [38].

### 2.5. Bacterial Cytotoxicity Tests

Moreover, the cytotoxicity due to supplement of ChNPs and exposure to PEF for each bacterial strain was evaluated by measuring levels of LDH, protein leakage and nucleic acid leakage in the broth media. The LDH levels were obtained by monitoring changes in supernatant turbidity. The protein leakage levels were measured by the Bradford method [39,40] using Coomassie protein assay reagent (Pierce, Rockford, IL, USA) as adapted by Li et al. [41]. The nucleic acid leakage was evaluated by measuring the amount of acid released in the media as adopted by Reddy et al. [42]. The cytotoxicity parameters of LDH, protein leakage, and nucleic acid leakage were evaluated relative to the negative control samples by using a microplate spectrophotometer system (Spectramax190, Molecular Devices, San Jose, CA, United States) at 490 nm, 595 nm and 260 nm, respectively. The data is represented in relative percentages as follows:

Parameter% = [(treated samples − control samples)/control samples] × 100.

### 2.6. Statistical Analysis

We designed the experiments based on three replicates. Each replicate has 10 samples per each treatment condition (i.e., the total number of samples is 30 per treatment). Then we averaged the results of these 10 samples to get one result/replicate and used these three results/treatment to perform statistical analysis. The statistical analysis was executed by using the SPSS package version 21 (IBM, Armonk, NY, USA) for Windows and all data was presented in terms of mean values ± standard deviation (SD). Furtherly, the data were statistically evaluated by one-way analysis of variance (ANOVA) to determine the significance of differences between results. The differences were considered by the post hoc Tukey test to be statistically significant at *p* < 0.05 and statistically highly significant at *p* < 0.01.

## 3. Results and Discussion

**Figure 2:**
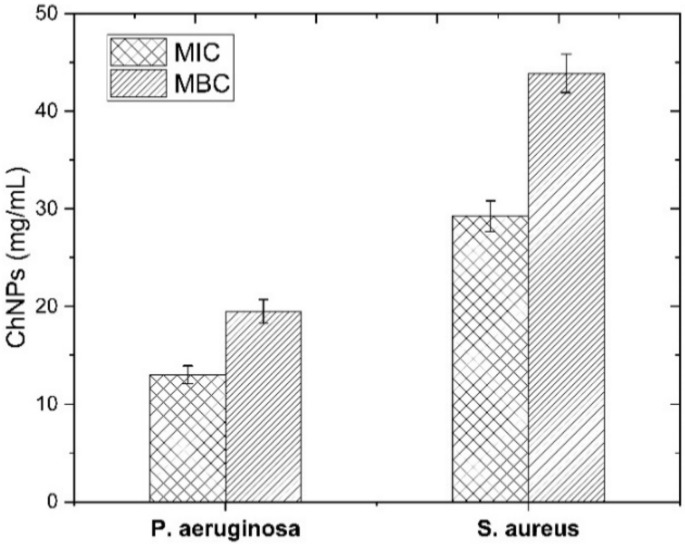
MIC and MBC levels of bacteria strains after supplementing with ChNPs at different concentrations.

The bacteriostatic and bactericidal effects of ChNPs were monitored against both bacteria models as tabulated in Table 1, whereas MIC and MBC levels were obtained as shown in Figure 2.

The bacteriostatic levels indicated a higher concentration (29.3 mg/mL) for *S. aureus* than for *P. aeruginosa* (13.1 mg/mL), which reflects the greater potential activity of ChNPs against Gram negative bacteria than positive ones. In the same way, levels of ChNPs that caused > 99.9% of bacteria to be killed indicated a supplementation of 19.5 mg/mL and 43.9 mg/mL of ChNPs against *P. aeruginosa* and *S. aureus*, respectively. There are multiple possible interactions between ChNPs and bacteria. The antibacterial activity of ChNPs resulted from its polycationic structure due to the protonation of –NH_2_ groups on the C-2 position of the D-glucosamine repeat unit as reported elsewhere [43,44], so the interaction between positively charged ChNPs with negatively charged bacteria cell surface deactivates the normal functions of the membrane. The deactivation happens either by enhancing the leakage of intracellular components or by damping the transport of nutrients into cells [45]. The dysfunction of the bacterial cell membrane leads to changes in cell permeability that allow the ChNPs to enter inside the cell and so, it may bind to DNA and disrupt its replication, which in turns leads to bacterial cell death [46]. Comparing Gram negative and positive bacteria, ChNPs show variable interaction mechanisms based on the differences in the cell wall and cell membrane morphological structure of both types. Our findings resemble results reported elsewhere [47,48,49,50,51] and confirm the higher effect of ChNPs against Gram negative bacteria than positive ones. The cell membrane structure of Gram negative bacteria contains lipopolysaccharide anionic groups (phosphate and pyrophosphate groups) that supply more negative charges to the cellular outer surface. Such an excess of static negative charges at the cell surface allows ChNPs to interact more strongly with Gram negative stained bacteria than positive ones [52,53,54].

The aim of the current study was to enhance the impact of ChNPs as an antibacterial agent and therefore this work was extended to evaluate their combination with exposure to PEF. It is known that the influence of external PEF is dependent on the physical field conditions such as field strength, applied frequency and exposure time, so it is worth stating that the exposure to our specific field conditions (50% duty cycle, 3.70 ± 0.18, kV/m, 30 min at 0.7 Hz and 20 Hz) has non-ionizing and non-thermal effect and has a very low encounter energy. Therefore, it is proposed not to cause any specific molecular changes in the ChNPs structure and/or any cellular ionic constituents [55,56,57].

**Figure 3:**
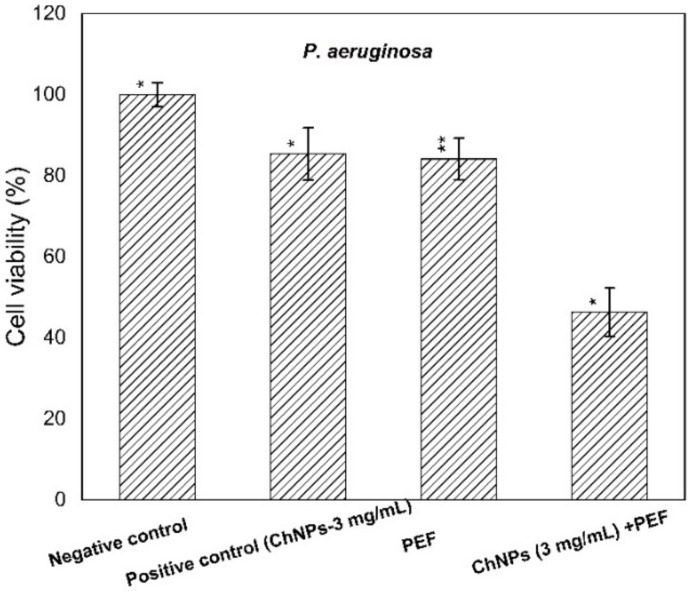
Cell viability percentages of *P. aeruginosa* relative to control bacterial counts. * statistically significant, ** statistically highly significant.

**Figure 4:**
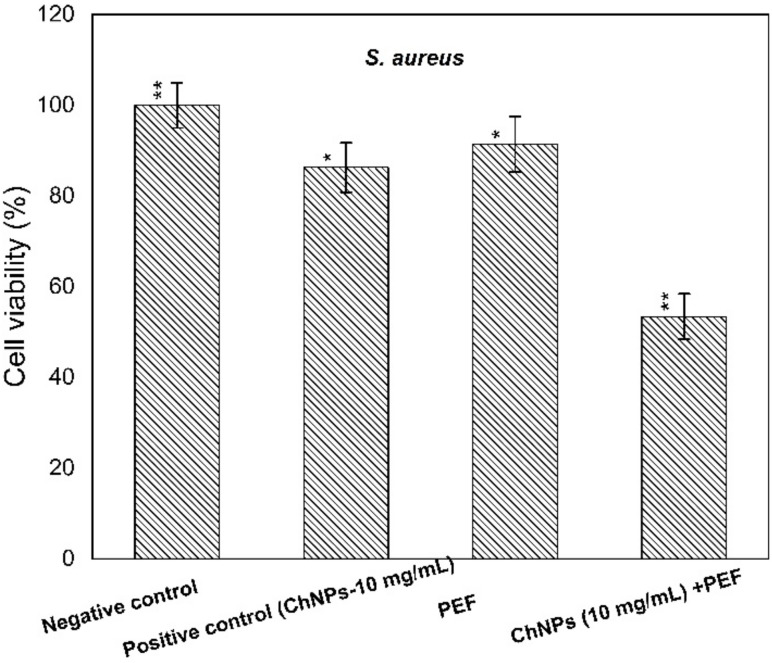
Cell viability percentages of *S. aureus* relative to control bacterial counts. * statistically significant, ** statistically highly significant.

The supplement of ChNPs was adapted at levels of 3 mg/mL and 10 mg/mL, lower than the MIC levels for *P. aeruginosa* and *S. aureus*, respectively. The cell viability percentages of supernatants treated with ChNPs, PEF, and combinations of them were calculated in comparison to control ones as depicted in both Figure 3 and Figure 4.

The obtained results indicated a remarkable antibacterial enhancement of the ChNP activity when combined with PEF compared to using ChNPs alone, by 40% and 30% for *P. aeruginosa* and *S. aureus*, respectively. One can explain the direct interaction of exposure to PEF based on the physics of the field-charge system. Of note the electric fields have the ability to drive, accelerate and orient charges under the field gradient, so electric field application may cause charges at the outer cell membrane to be dislocated. The combination of PEF exposure represents an extra cofactor to enhance ChNP activity not only at the cell surface but also to be driven more inside the cell and attach to DNA.

**Figure 5:**
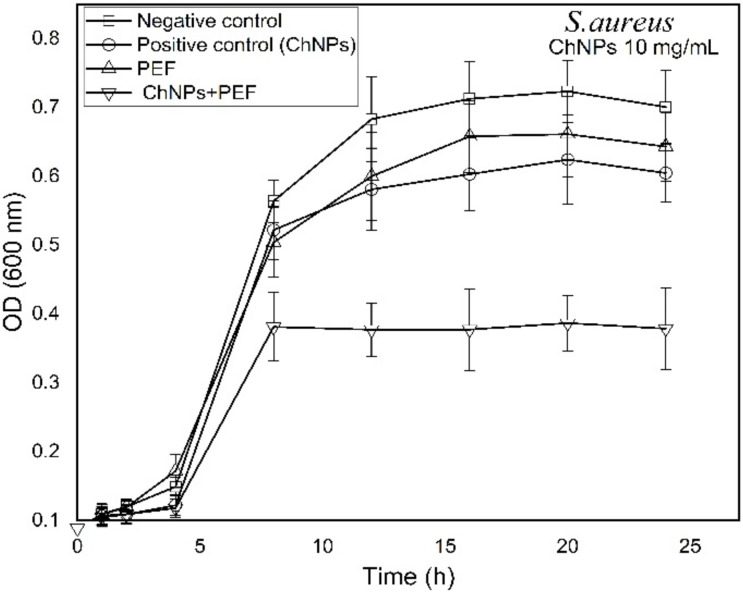
Growth time-dependent characteristic curves of *P. aeruginosa* as treated by ChNPs (3 mg/mL) and PEF each alone and combined with exposure to PEF.

**Figure 6:**
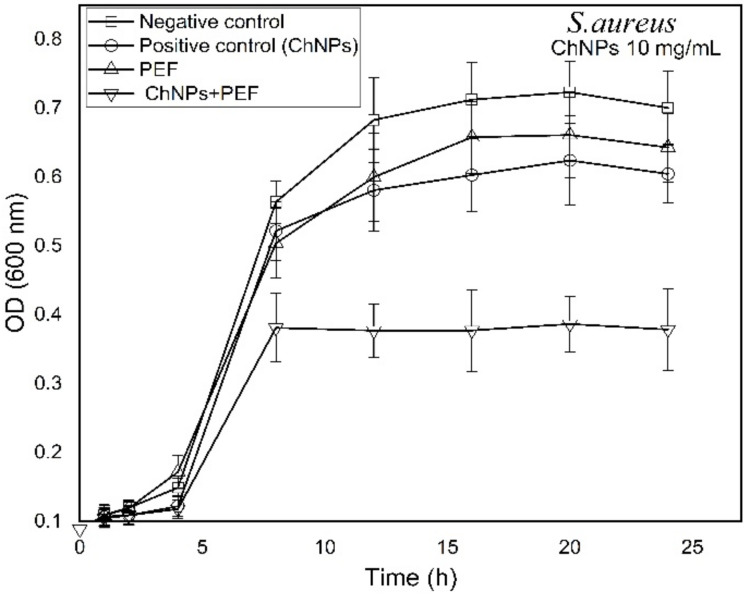
Growth time-dependent characteristic curves of *S. aureus* as treated by ChNPs (10 mg/mL) and PEF each alone and combined with exposure to PEF.

Growth-time dependent changes were observed by recording the OD (600 nm) every hour for samples treated by ChNPs alone and combined with exposure to PEF as shown in Figure 5 and Figure 6 for *P. aeruginosa* and *S. aureus*, respectively. The recorded data reflected the population density of bacterial cells within the growing broth media. It is known that as bacteria grow rapidly, the incident light through the broth scatters more and the OD values become higher. The growth curves shown in Figure 5 and Figure 6 depict similar dispersions of curves for all tested samples that represent three growth phases (lag, log and stationary). Significantly, the growth of *P. aeruginosa* cells was affected by the supplement of ChNPs in a way it had delay in the cell reproduction in the growing phases and the curves tend to be shallower than control ones as shown in Figure 5. Exposure to PEF showed an unstructured effect that started with as a supportive cofactor that enhanced the growth of bacteria till the 10th h of incubation and was then followed with a disruptive reaction that inhibited bacterial growth. The combination of exposure to PEF and supplementation with ChNPs provided the most powerful weapon against bacteria that caused the highest delay of bacterial replication and least bacteria population in the stationary phase. In addition, *S. aureus* growth curves presented in Figure 6 show almost the same growing population till the 8th h of incubation as compared to the control except for the samples treated with a combination of ChNPs and PEF. Like the Gram negative bacteria, a remarkable disruptive influence of ChNPs and PEF against *S. aureus* growth was observed even in the lag phase. Such an effect confirms the abnormalities in cellular metabolic activity accompanied by alterations in the bacteria cell division process. In particular, the combination of PEF and ChNPs shows a remarkable progression in its cell uptake and so drives more charged ChNPs inside the cell to hinder the DNA formation and stop the ability to replicate [58].

**Figure 7:**
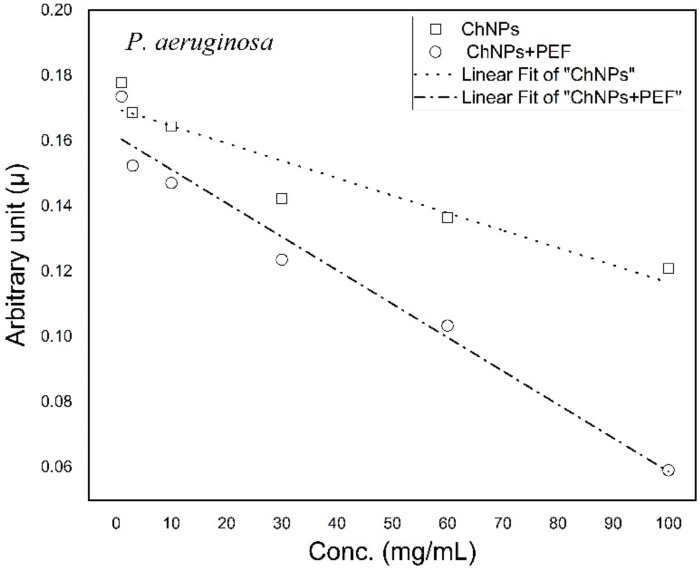
Trend lines of arbitrary constant values for *P. aeruginosa* treated by ChNPs alone and combined with PEF.

**Figure 8:**
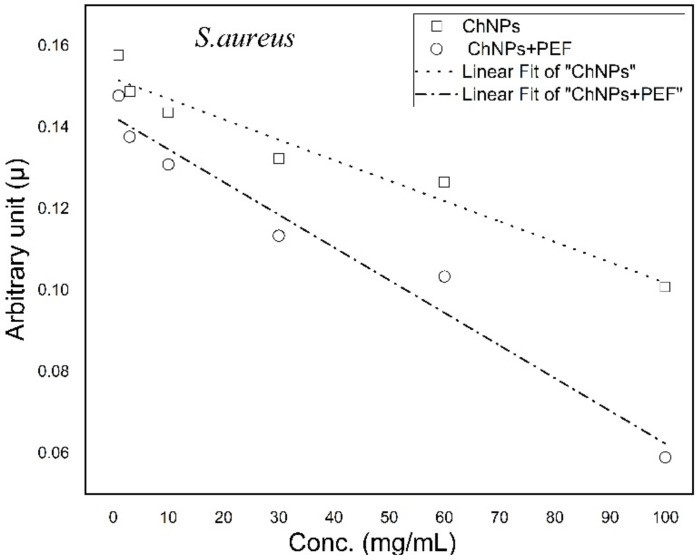
Trend lines of arbitrary constant values for *S. aureus* treated by ChNPs alone and combined with PEF.

Growth curves of each bacterial strain were analyzed and the growth rates were calculated for samples treated by ChNPs alone and combined with PEF. Then, arbitrary growth rate constants were graphed versus concentrations of ChNPs. Both Figure 7 and Figure 8 show the trend lines of the rate constants for *P. aeruginosa* and *S. aureus*, respectively. The trend lines of both bacterial samples treated by ChNPs combined with PEF showed significantly more diversity than with ChNPs alone. The trend lines of combined samples showed that the ChNP concentration dependency behevaed in a manner where as the concentration increases, the lines become more tilted. The slopes of arbitrary growth rate constants were calculated and are tabulated in Table 2. The influence of exposure to PEF showed the synergistic antibacterial impact of ChNPs as its combination displayed negative slope value increases by 90% and 60% for *P. aeruginosa* and *S. aureus*, respectively.

**Figure 9:**
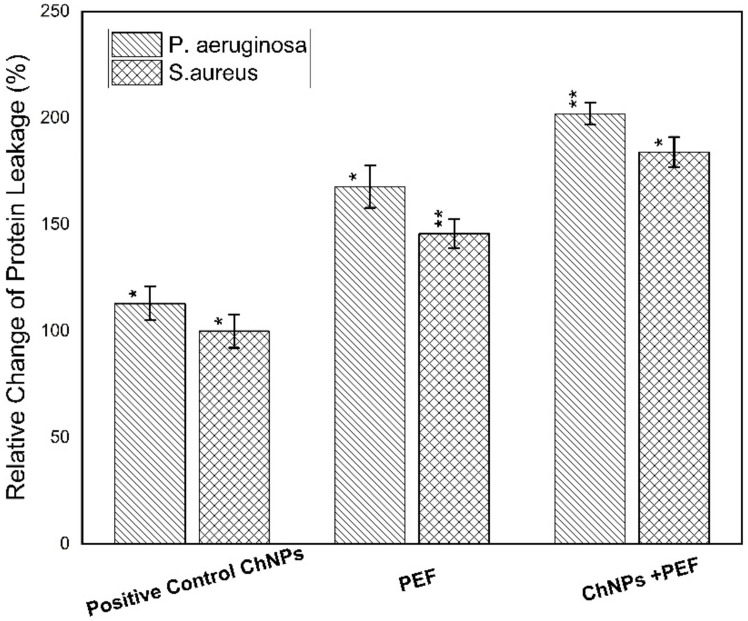
Relative change of protein leakage percentage for *P. aeruginosa* and *S. aureus* samples treated by ChNPs and PEF each alone and combined with exposure to PEF. * statistically significant, ** statistically highly significant.

**Figure 10:**
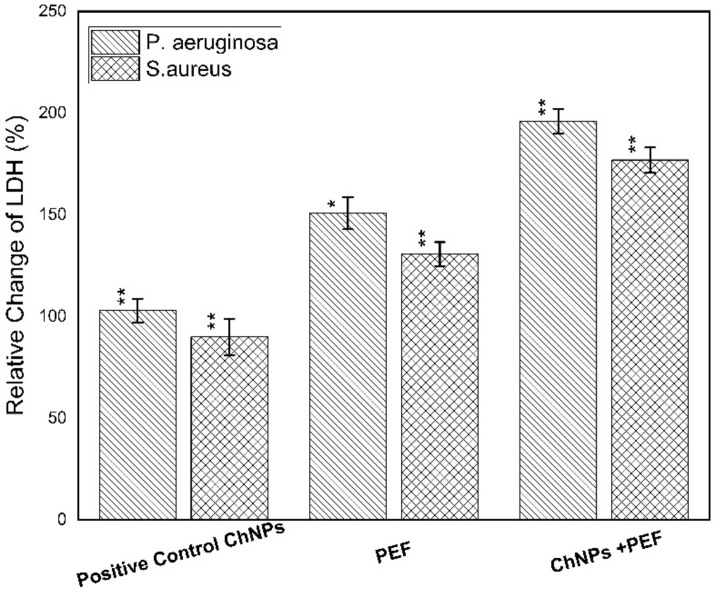
Relative change of LDH percentage for *P. aeruginosa* and *S. aureus* samples treated by ChNPs and PEF each alone and combined with exposure to PEF. * statistically significant, ** statistically highly significant.

Furthermore, the cytotoxicity towards the bacterial strains was obtained by measuring the levels of LDH, protein leakage and nucleic acid. The quantitative evaluation of LDH and protein leakage percentages relative to the negative control samples is considered as an effective biomarker for bacterial cell membrane damage. The loss of cell membrane integrity as a result of treatment has been manifested by its leakage from the inside of bacteria into the extracellular medium [59]. Both Figure 9 and Figure 10 show the relative change in percentages of protein leakage and LDH values for *P. aeruginosa* and *S. aureus* samples treated by ChNPs and PEF each alone and combined with exposure to PEF.

**Figure 11:**
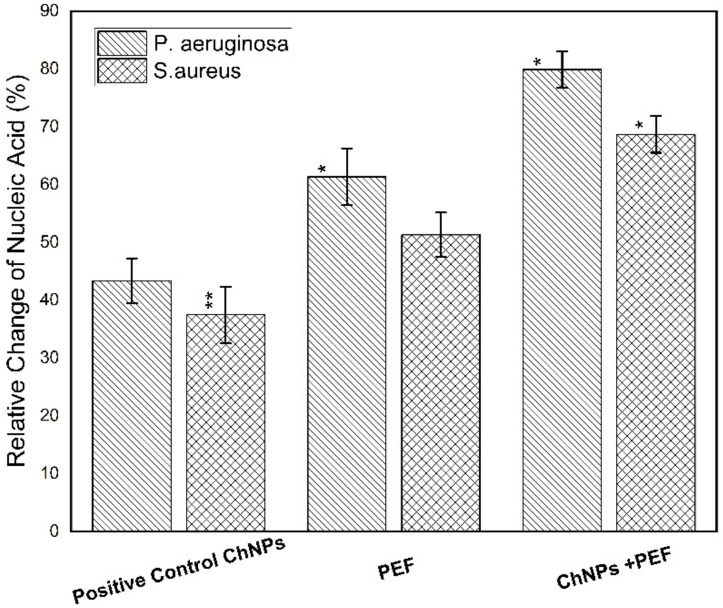
Relative change of nucleic acid percentage for *P. aeruginosa* and *S. aureus* samples treated by ChNPs and PEF each alone and combined with exposure to PEF. * statistically significant, ** statistically highly significant.

Of note, the leakage of intercellular macromolecules such as proteins outside the cell demonstrates the increase in membrane permeability. Our findings validate the highest effect of treatment by both ChNPs and PEF than for each one alone for *P. aeruginosa* and *S. aureus* on the order of (180 µg/mg-ChNPs + PEF) > (168 µg/mg-PEF) > (157 µg/mg-ChNPs) and (160 µg/mg-ChNPs + PEF) > (146 µg/mg-PEF) > (129 µg/mg-ChNPs), respectively. Moreover, the release of LDH enzyme into the surrounding extracellular space only happens when cell membrane integrity is compromised. When LDH is present in the cell culture, it reduces NAD^+^ to NADH and H^+^ through the oxidation of lactate to pyruvate. Afterward, the catalyst (diaphorase) then transfers H/H^+^ from NADH + H^+^ to the tetrazolium salt INT to form the red-colored formazan salt. Based on this standard, the colorimetric absorbance is proportional to the amount of damaged cells in the culture. The LDH levels were normalized to the negative control samples, averaged and represented as percentages. The *P. aeruginosa* data indicated elevations by 1-fold (380 µU/mL-ChNPs), 1.5-fold (475 µU/mL-PEF) and 2-fold (570 µU/mL-ChNPs + PEF) compared to control values as shown in Figure 10. Similarly, the *S. aureus* data showed an increase of LDH levels by < 1-fold (170 µU/mL-ChNPs), ~1-fold (425 µU/mL-PEF) and ~1.5-fold (570 µU/mL-ChNPs + PEF) compared to control ones as shown in Figure 10. Due to the inherent linearity of this LDH cytotoxicity assay, it may be presumed that the percentage of cellular damage of samples treated by ChNPs and PEF represents the highest injury level. Collectively, our results show that the treatment with ChNPs in combination with PEF remarkably disrupted the bacterial membranes, consequently leading to intracellular leakage of macromolecules. The exposure to PEF has the ability to cause outer charges to be disordered and so, the binding of even small amounts of ChNPs to the cellular outer surface has an additional bacteriostatic action compared to using ChNPs alone. Not only this, but it also has a bactericidal effect resulting from the ability of more ChNPs to enter the cell and combine directly into DNA and hinder its replication ability. Nucleic acids have a UV absorption maximum at a wavelength of 260 nm, and the concentration of nucleic acid was directly proportional to the OD value. The absorbance values might in part be due to the release of nucleic acid-related molecules, such as nucleotides, or even to proteins [60] so it is supposed that there is a direct relationship between bacterial nucleic acid leakage and bacterial inactivation. The OD values confirmed the highest effect of treatment by ChNPs combined with PEF as shown in Figure 11. The synergistic combination effect is depicted in the order of OD_260_ as follow; (0.98-ChNPs + PEF) > (0.78-PEF) > (0.49-ChNPs) and (0.91-ChNPs + PEF) > (0.73-PEF) > (0.58-ChNPs) for *P. aeruginosa* and *S. aureus*, respectively.

**Figure 12:**
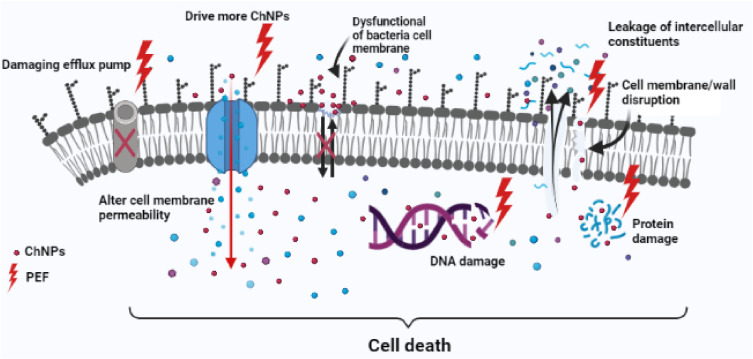
Schematic diagram of possible antimicrobial synergism of ChNPs and PEF against bacterial cell.

The use of PEF may have a specific cellular response against DNA synthesis, transcription, and protein synthesis [61,62,63,64], so our suggestion is to take advantage of using PEF as a wound healing accelerator and its synergistic effect as an antibacterial enhancer. PEF has a direct impact on the wound healing process by altering or augmenting preexisting endogenous electrical fields. It may also have multiple mechanisms of action that help in angiogenesis and increase collagen synthesis in addition to its bactericidal effect [64,65]. From this standpoint, the importance of our new combination lies in the use of safe nanomaterials such as chitosan with unique characteristics and exposure to PEF of low frequencies with non-thermal and non-harmful effects. Summing up, all the previous possible antimicrobial mechanisms of ChNPs and PEF are represented in schematic diagram to show how it may work effectively against *P. aeruginosa* and *S. aureus* are shown in Figure 12. 

## 4. Conclusions

Our data confirms the synergistic antibacterial effect of ChNPs and exposure to PEF against *P. aeruginosa* and *S. aureus*. Specifically, the antibacterial impact of exposure to ChNPs and PEF is found to be higher against *P. aeruginosa* than *S. aureus* bacteria. Such a new combination with PEF has a positive impact not only by accelerating the wound healing process but also by acting as an antibacterial cofactor with ChNPs. These results are promising and could be extended in the future to examine several other types of bacteria and the application in vivo along with all treatment conditions should be studied.

## Figures and Tables

**Table 1:** List of *P. aeruginosa* and *S. aureus* counts (CFU/mL ± SE) under supplement of ChNPs at different concentrations.

Conc. of ChNPs (mg/mL)	Count of *P. aeruginosa* (CFU/mL)	Count of *S. aureus* (CFU/mL)
65.8	NG	NG
43.9	NG	NG
29.3	NG	(7.77 ± 0.62) × 10^1^
19.5	NG	(4.79 ± 0.51) × 10^7^
13.1	(3.09 ± 0.13) × 10^1^	(2.91 ± 0.33) × 10^8^
10.6	(7.76 ± 0.18) × 10^3^	(8.51 ± 0.11) × 10^8^
5.78	(1.62 ± 0.26) × 10^6^	(1.28 ± 0.12) × 10^9^
3.19	(4.07 ± 0.43) × 10^7^	(2.81 ± 0.31) × 10^9^
1.71	(8.71 ± 0.35) × 10^8^	(5.37 ± 0.22) × 10^9^

CFU: colony forming units, NG: No Growth.

**Table 2:** List of slope trend line values of arbitrary growth rate constants *P. aeruginosa* and *S. aureus* treated by ChNPs alone and combined with PEF.

Treatment Condition	Slope of Arbitrary Constants (µ) × 10^−4^ *P. aeruginosa*	Slope of Arbitrary Constants (µ) × 10^−4^ *S. aureus*
ChNPs	−(5.33 ± 0.86)	−(5.02 ± 0.56)
ChNPs +PEF	−(10.3 ± 0.96)	−(8.03 ± 0.75)
